# Case of Imperforate Rectum

**Published:** 1853-09

**Authors:** D. C. Dewey

**Affiliations:** N.Y. Mills


### ART. II.— *Case of Imperforate Rectum.* By D. C. Dewey, M. D.

Messrs. Editors,—I give below the outlines of a case which has recently occurred in my practice, and which you are at liberty to publish, if you consider it of sufficient interest to be presented to your readers:

I was called, on the 12th inst., to attend Mrs. P. in her first confinement. After a somewhat tedious and protracted labor, she was delivered of an apparently healthy and well-formed male child, about 4, P. M.

The child at first took the breast readily, but after a few hours refused it, and was evidently drooping.

At the period of thirty-six hours from birth, there had been but a slight flow of urine, and no discharge of the meconium, although the usual measures had been resorted to by the nurse, under my directions, to procure the ordinary evacuations.

I now attempted personally to use an enema, but found it would not pass up. Suspecting immediately a closure of the canal near the anus, I introduced an instrument and found myself unable to carry it more than 1½ in. beyond the external outlet.

Having satisfied myself of the closure, and being unwilling, under the circumstances, to take any further steps entirely upon my own responsibility, Dr. H. was sent for to advise with me, but being absent from home, Dr. S. called.

Dr. S. recommended a resort to no active measures further than exerting with a blunt instrument as much force as would be safe in attempting to open the passage, proceeding in this manner, in view of the possibility of an adhesion having occurred between the opposed inner walls of the rectum, of a limited extent and not sufficient firmness to withstand such measures.

Dr. S. fell in with a suggestion made by me, that as a *dernier* resort it might be well to attempt to open the passage by means of a cutting instrument introduced through the anus.

The above-mentioned consultation was had in the morning. In the afternoon I met Dr. H. Dr. H. advised immediately following out the suggestion I had made in the morning, and therefore with my concurrence, introduced a large sized trocar as nearly as possible in the natural course of the rectum. After cutting, as he thought, half or three-quarters of an inch, he reached the pervious bowel above, and upon withdrawing the trocar, a considerable quantity of meconium, with flatus, passed off through the canula. This was attended with considerable diminution of the abdominal distention and evident relief to the little patient.

An enema was now administered, also a dose of castor-oil, and directions given for enema to be renewed if an evacuation should not take place within a few hours.

At the time of the operation not much bleeding was apparent, but a few hours afterward, about 9 or 10, P. M., I was summoned on account of a considerable hemorrhage which was then proceeding. I succeeded in soon checking this by the use of astringents, but upon searching for the artificial passage could not find it, neither was I able to throw up an injection.

The next morning, with a blunt instrument, I again endeavored, but without success, to reopen it. About 6, P. M., the child died.

I made, the next morning, a post-mortem examination and found the obstruction, as before stated, not more than one and a half inches from the anus. At this point the rectum was constricted, taking the appearance of a solid cord, about half an inch in diameter, its canal being entirely obliterated for a distance of about three-quarters or half an inch. The trocar had traversed with accuracy this solid substance, so as not to penetrate the peritoneal cavity, but the incision appeared to have been commenced in one of the walls of the anal cavity near its base, and on this account was probably more ready to close and more difficult to be reopened.

The question now arises: Could the life of the child have been saved under any plan of treatment? This I will not presume to answer, but should another apparently similar case occur to me, I think I should prefer to pursue something like the following course:

1st. Operate early and in the manner pursued in the present instance.

2d. Take immediate measures to check or prevent hemorrhage.

3d. Introduce a tube and keep it in position in order to prevent obliteration of the passage.

4th. Dilate, from time to time, by means of bougies, as vigorously as safety would admit, introducing larger tubes till a sufficient caliber should be obtained, and the danger of closure should be past.

These propositions are founded upon the supposition of the physical condition being the same, which could not be positively determined beforehand. Should it, however, prove to be so, I should hope for a favorable result.

N. Y. Mills, July 26, 1853.

